# High throughput sequencing sheds light on the viral diversity in *Aedes aegypti* mosquitoes from Binh Thuan Province, Vietnam

**DOI:** 10.1371/journal.pone.0322924

**Published:** 2025-05-23

**Authors:** Margarita Popova, Anna Gladkikh, Alena Sharova, Tatiana Arbuzova, Valeriya Sbarzaglia, Ekaterina Klyuchnikova, Nadezhda Tsyganova, Majid Forghani, Anastasia Gritseva, Edward Ramsay, Nguyen Thanh Dong, Bui Thanh Phu, Do Thai Hung, Vladimir Dedkov

**Affiliations:** 1 Saint Petersburg Pasteur Institute, Saint Petersburg, Russia; 2 Pasteur Institute in Nha Trang, Vietnam; 3 Martsinovsky Institute of Medical Parasitology, Tropical and Vector Borne Diseases, Sechenov First Moscow State Medical University, Moscow, Russia; Faculdade Sao Leopoldo Mandic, BRAZIL

## Abstract

Mosquitoes are important vectors for various infectious pathogens. More than 200 species of mosquitoes are common in Vietnam, one of the main carriers of viruses that are important for humans is the mosquito of the genus *Aedes aegypti*. Metavirome sequencing can shed light on the diversity of mosquito-borne viruses classified as insect-specific viruses (ISV). After BLAST analysis using the viral database, contigs were classified as belonging to seventeen ISVs. Ten of them are distributed among five families: *Totiviridae*; *Flaviviridae*; *Partitiviridae*; *Phenuiviridae;* and *Orthomyxoviridae*. The other seven species belonged to recently identified RNA viruses whose taxonomic position is undefined in the current classification of the International Committee on Taxonomy of Viruses (ICTV). This is the first study to reveal the diversity of RNA viruses associated with *Aedes aegypti* mosquitoes in Vietnam, while highlighting the need for further study of ISV in mosquito vectors.

## 1. Introduction

Mosquitoes are vectors of several common and emerging mosquito-borne diseases, specifically chikungunya, dengue, Japanese encephalitis, malaria, West Nile, yellow fever, and Zika. According to the assessment of some experts, half of the world’s population is under the risk of viral diseases vectored by mosquitoes, causing death in millions of people annually [1]. Increased international travel leads to the rapid spread of viral infections, as well as an increased risk of mutation in pathogen genomes [2]. Among mosquito-borne diseases (MBD), dengue fever is the most common infectious disease transmitted by *Aedes aegypti* mosquito, which breeds around domestic or human habitations [3,4].

About half of the world’s population is now at risk of dengue fever with an estimated 100–400 million infections occurring each year. The incidence of dengue fever has grown dramatically around the world in recent decades. Cases reported to the WHO increased from 505,430 cases in 2000 to 5.2 million in 2019. The disease is now endemic in more than 100 countries. The WHO Region of the Americas reported 4.5 million cases in 2023, with 2300 deaths. High case numbers were reported in Asia: Bangladesh (321,000); Malaysia (111,400); Thailand (150,000); and Vietnam (369,000) [5].

Vietnam is a tropical country located in Southeast Asia wherein the population is at high risk of acquiring mosquito-borne diseases such as malaria, dengue fever, Zika fever, Japanese encephalitis, and lymphatic filariasis [6]. At least 281 species of mosquito have been reported to be common in Vietnam, of which the main vectors of zoonotic disease are mosquitoes belonging to genera *Aedes*, *Anopheles,* and *Culex* [7].

Over the past two decades, an increasing number of arthropod-specific viruses, classified as insect-specific viruses (ISV), have been identified in various mosquito populations around the world [8]. Insect-specific viruses are highly prevalent in wild mosquito populations and have been reported to suppress, enhance, or have no effect on replication of medically important arboviruses, potentially affecting vector competence. There is a potential use of ISVs as biocontrol agents. The close genetic similarity between ISVs and arboviruses has the potential for interference in their replication, either through upregulation of antiviral immune responses in the vector, or via superinfection exclusion (i.e., homologous interference), wherein similar viruses can block each other through competition [9].

Metavirome sequencing of mosquitos has led to an exponential increase in the number of mosquito-borne viruses identified over the past couple of years and has provided new insight into the enormous complexity and diversity of invertebrate RNA viruses [10]. In Vietnam, metagenomic sequencing serves several purposes: support of prevention efforts; understanding ongoing dengue fever transmission and its etiology; and providing data for real-time public health interventions. Together, these facilitate accurate diagnosis, timely treatment, and disease control [11]. Studying the virome of *Aedes* mosquitoes, the most common vector of dengue virus in Vietnam, may enable a more accurate assessment of mosquito-borne disease risk, vector competence, and mosquito control measures.

## 2. Materials and methods

### 2.1. Mosquito samples and preparation

**Fig 1:**
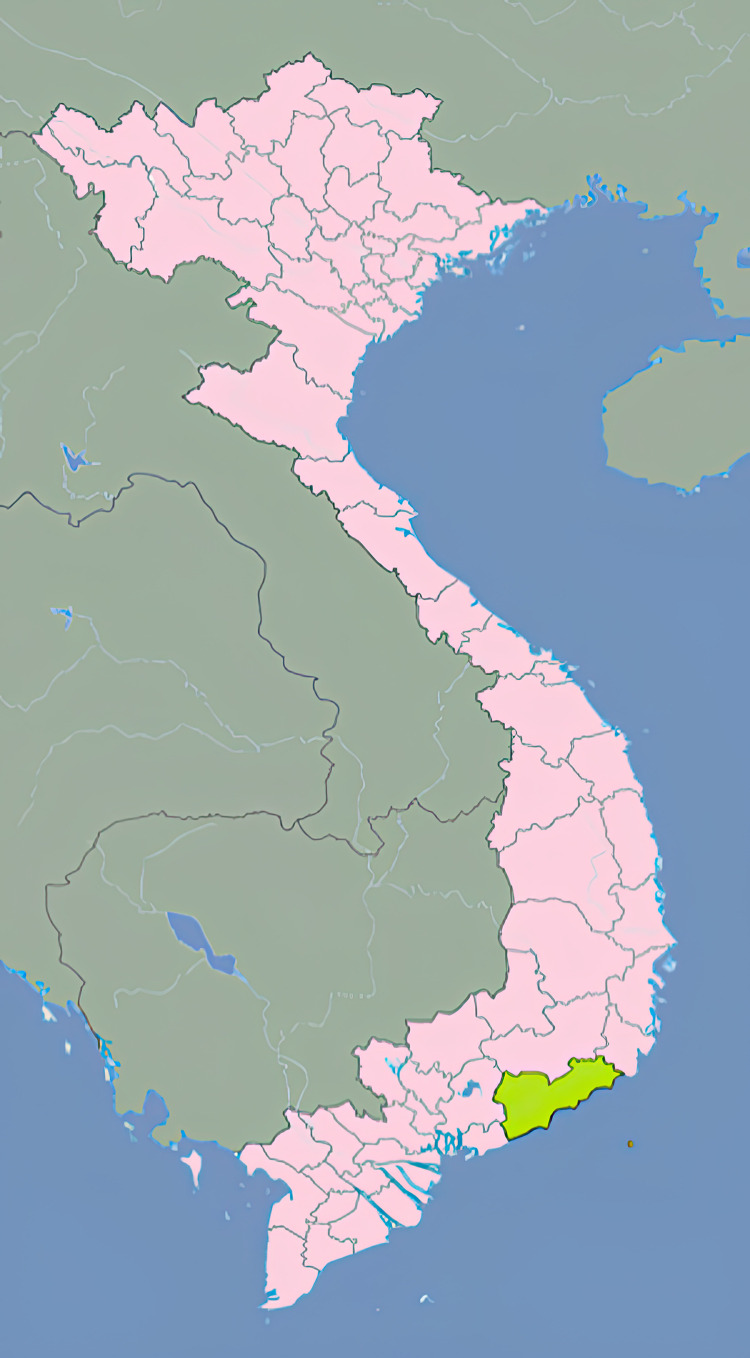
Binh Thuan Province, Vietnam.

Mosquitoes were collected from Binh Thuan Province, Central Vietnam (Fig 1). Mosquito larvae and pupae were collected from water near residential houses from both small and large containers (tanks, ponds, etc.) [12]. Next, larvae were transported to the laboratory of the Pasteur Institute in Nha Trang. Larvae were reared to adult stage according to technical guidelines for larval and mosquito rearing at the Department of Entomology laboratory [13]. After development of pupae into adults, mosquito classification was carried out in accordance with the Illustrated Key to Mosquitoes of Vietnam by Chester J. Stojanovich and Harold George Scott [14]. The classification process was conducted at the Laboratory of the Entomology (Pasteur Institute Nha Trang).

The province in the territory of Vientiane where the samples were collected. Reprinted from [https://commons.wikimedia.org/wiki/File:Location_of_Binh_Thuan_within_Vietnam.png] under a CC BY license. The drawing is similar, but not identical, to the original image and is for illustrative purposes only.

For the study, 70 female *Aedes aegypti* (*Ae. aegypti*) mosquitoes were selected in the laboratory, and subsequently pooled into microcentrifuge tubes (10 individuals per pool). Male mosquitoes were removed to focus on viruses that could be transmitted to animals.

Collected samples were washed in 70% ethanol to prevent surface contamination and rinsed twice in a sterile phosphate-buffered saline (PBS) solution (Sigma-Aldrich, Selangor, Malaysia). Each mosquito pool was homogenized in 2 ml microcentrifuge tubes (Eppendorf, Hamburg, Germany) containing two 5-mm diameter stainless steel beads (QIAGEN, Hilden, Germany) and 400 microliters (μL) of 1 × PBS (Sigma-Aldrich, Selangor, Malaysia). Homogenization was performed using a TissueLyser LT homogenizer (QIAGEN, Hilden, Germany). The supernatant (400 μL) was transferred into 2 ml microcentrifuge tubes for further RNA isolation and storage.

### 2.2. RNA extraction and quantification

RNA samples were obtained by extraction and purification using the QIAamp® Viral RNA Extraction Kit® (QIAGEN, Hilden, Germany) with the QIAcube Connect automatic station (QIAGEN, Hilden, Germany) according to manufacturer recommendations. The RNAs were eluted with 50 µL of AVE Buffer® (QIAGEN, Hilden, Germany) and stored at −70°C until further analysis. The concentrations of extracted RNA samples were measured using a Qubit 4 fluorimeter (Thermo Fisher Scientific, Massachusetts, USA) and the Qubit RNA HS Assay Kit (Invitrogen, Waltham, MA, USA). When measuring the amount of total RNA, results were obtained in the range of 8–12 ng. The amounts of total RNA for each sample are presented in Table 1.

**Table 1:** Amount of input RNA for library preparation.

Sample Name	Input RNA, nanograms (ng)
BTMP1	8.5
BTMP2	9.2
BTMP3	8.9
BTMP4	11.9
BTMP5	10.5
BTMP6	11.1
BTMP7	9.9

### 2.3. Library preparation

KAPA RNA HyperPrep Kit reagents (Roche, Mannheim, Germany) were used to prepare libraries. Libraries were prepared according to manufacturer recommendations including several steps: fragmentation and priming reaction; synthesis of the first strand cDNA; synthesis of the second strand cDNA and A-tailing; and adapter ligation (KAPA Universal Adapter, Roche, Mannheim, Germany). After adapter ligation, samples were purified with KAPA Pure Beads. The resulting libraries were then amplified and again cleaned using KAPA Pure Beads (Roche, Mannheim, Germany) according to manufacturer recommendations.

For quality control, 10 ng of purified libraries were used for capillary electrophoresis in the QIAxcel Advanced System (QIAGEN, Hilden, Germany). Fragment distribution of libraries was in the range 190–450 bp, with a median of 220–270 bp. All libraries were quantified using a Qubit 4.0 Fluorometer (Thermo Fisher Scientific, Massachusetts, USA) and Qubit dsDNA HS Assay Kit (Invitrogen, Waltham, MA, USA) prior to sequencing. Sequencing was performed on a MiSeq (Illumina, Inc., California, USA) instrument using MiSeq Reagent Kit v3 with paired 300 bp reads (Illumina, California, USA).

### 2.4. Contig assembly and BLAST analysis

Quality control was performed on the raw sequence data using FastQC (v0.12.1) [15] to evaluate read quality and to confirm data integrity. Low-quality reads and adapters were trimmed using Trimmomatic (v0.39) applying sliding window trimming with a window size of four and a minimum average quality score of 20 [16]. The cleaned host-filtered reads were assembled *de novo* to construct viral contigs using SPAdes (v3.15.5) with the careful mode activated to improve assembly quality [17]. Viral identification of assembled contigs was performed using BLASTn (v2.14.0) against an NCBI viral nucleotide database, with results filtered for a minimum alignment length of 100 bases and limited to the top five hits per query.

### 2.5. Phylogenetic analysis

To confirm the phylogenetic relationship of the viruses identified in this study, all relative sequences for each species were retrieved from the GenBank database. Sequences were aligned by using the ClustalW method with the MEGA 11 software. For phylogeny reconstruction, sequences that have an overlap area were taken into analysis; the rest were excluded. Phylogenetic analysis was conducted in MEGA 11 using the maximum-likelihood method and the best-fit substitution model [18]. Final phylogenetic trees were generated after 1000 bootstrap replicates.

## 3. Results

**Fig 2:**
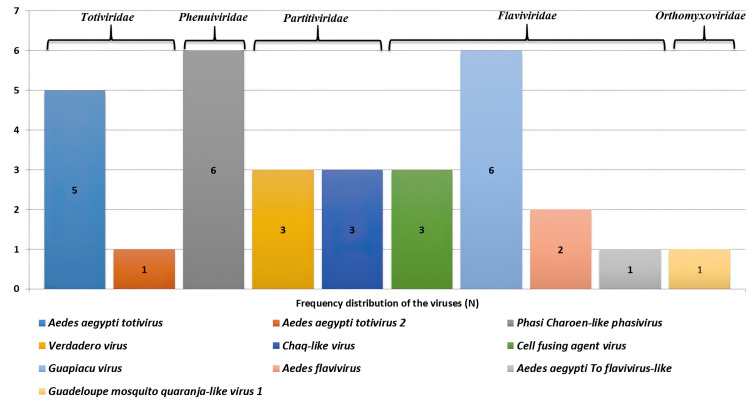
Frequency distribution of the viruses found in mosquito pools.

A total of seven NGS libraries were constructed and sequenced, resulting in 18.9 Gb of raw data, totaling 36 million reads. After quality control, 12.7 Gb of clean data and 34 million high-quality reads remained. From these, 150 viral contigs were obtained through *de novo* assembly of 16.3 million reads, accounting for 47% of total reads. The proportion of viral reads in each library was found to vary, reaching up to 68%. Following BLAST analysis against a viral database, the contigs were classified as belonging to 10 viruses distributed among five families: *Totiviridae* (with *Aedes aegypti totivirus* (AaTV) and *Aedes aegypti totivirus 2* (AaTV2)); *Flaviviridae* (including *cell fusing agent virus* (CFAV), *Guapiacu virus* (GUAPV), *Aedes flavivirus* (AEFV), and *Aedes aegypti To flavivirus-like* (AaTFLV)); *Partitiviridae* (featuring *Verdadero virus* and *Chaq-like virus*); *Phenuiviridae* (with *Phasi Charoen-like phasivirus* (PCLV)); and *Orthomyxoviridae* (represented by *Guadeloupe mosquito quaranja-like virus 1* (GMQLV1)), as depicted in Fig 2.

**Fig 3:**
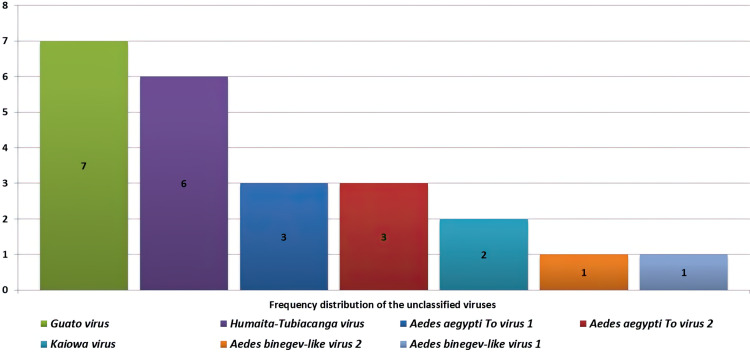
Frequency distribution of the unclassified viruses found in mosquito pools.

Another 76 contigs belonged to recently identified RNA viruses, the taxonomic positions of which are not defined in the current ICTV version: *Aedes aegypti To virus 1; Aedes aegypti To virus 2; Guato virus; Humaita-Tubiacanga virus* (HTV); *Kaiowa virus; Aedes binegev-like virus 1; and Aedes binegev-like virus 2* (Fig 3). According to the ICTV, *Kiowa virus* and *Guato virus* are related to the *Chuviridae* family of viruses. The viruses found in the samples were classified as insect-specific viruses (ISV). Arboviruses of medical significance were not identified in the analyzed samples.

**Fig 4:**
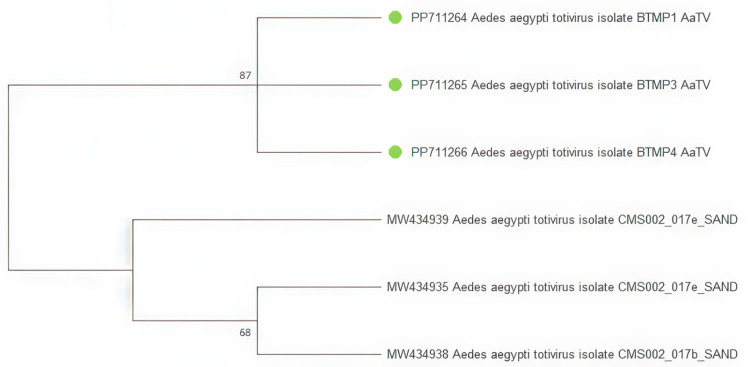
Phylogenetic bootstrap consensus tree of the *Aedes aegypti totivirus* (AaTV) fragment. The evolutionary history was inferred using the Maximum Likelihood method and Jukes-Cantor model [19]. Branches corresponding to partitions reproduced in less than 50% bootstrap replicates are collapsed. This analysis involved six nucleotide sequences. There were a total of 235 bp in the final dataset. The sequences obtained during the study are marked on the tree with green dots.

Fragments of the *Aedes aegypti totivirus* (family *Totiviridae*) were found in five samples. After alignment, three of the five fragments are 235 bp. were used to construct a phylogenetic tree. On the phylogenetic tree, isolates BTMP1_AaTV, BTMP3_AaTV, and BTMP4_AaTV are grouped into a separate cluster from isolates obtained from *Aedes aegypti* mosquitoes in California, USA (MW434939, MW434935, MW434938) (Fig 4).

**Fig 5:**
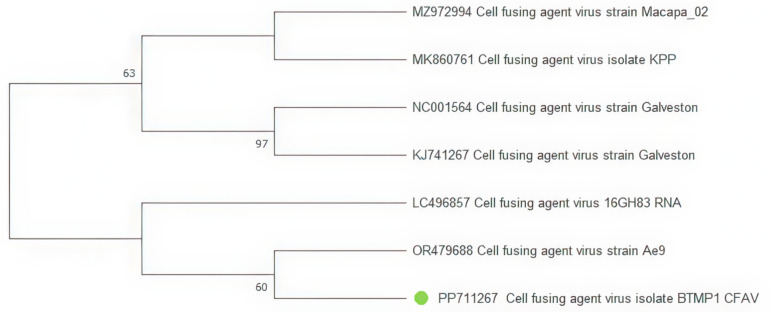
Phylogenetic tree of the *cell fusing agent virus* (CFAV) fragment. The evolutionary history was inferred using the Maximum Likelihood method and Kimura 2-parameter model [20]. Branches corresponding to partitions reproduced in less than 50% bootstrap replicates are collapsed. This analysis involved seven nucleotide sequences. There were a total of 370 bp in the final dataset. The sequences obtained during the study are marked on the tree with green dots.

Contigs of *cell fusing agent virus (*family *Flaviviridae)* were detected in three samples. After alignment, only one sequence was used to construct the tree due to the present common genome 370 bp fragment (Fig 5). On the dendrogram, the BTMP1_CFAV isolate is grouped into a separate cluster with isolates from Ghana (LC496857) and the Kingdom of Cambodia (OR479688).

**Fig 6:**
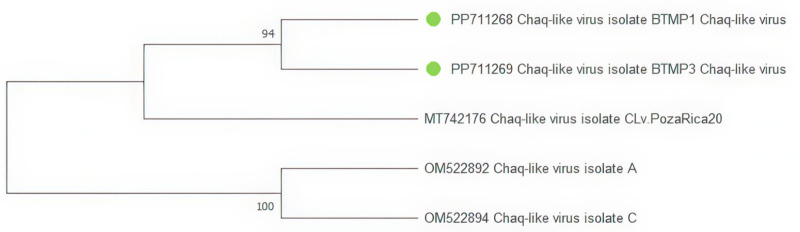
Phylogenetic tree of the *Chaq-like virus* fragment. The evolutionary history was inferred using the Maximum Likelihood method and Hasegawa-Kishino-Yano model [21]. Branches corresponding to partitions reproduced in less than 50% bootstrap replicates are collapsed. This analysis involved five nucleotide sequences. There were a total of 1375 bp in the final dataset. The sequences obtained during the study are marked on the tree with green dots.

Four contigs classified as *Chaq-like* viruses were found in three samples, and two sequences were used to construct the tree (Fig 6). The BTMP1_Chaq-like virus and BTMP3_Chaq-like virus isolates are grouped together next to a strain from Mexico (MT742176).

**Fig 7:**
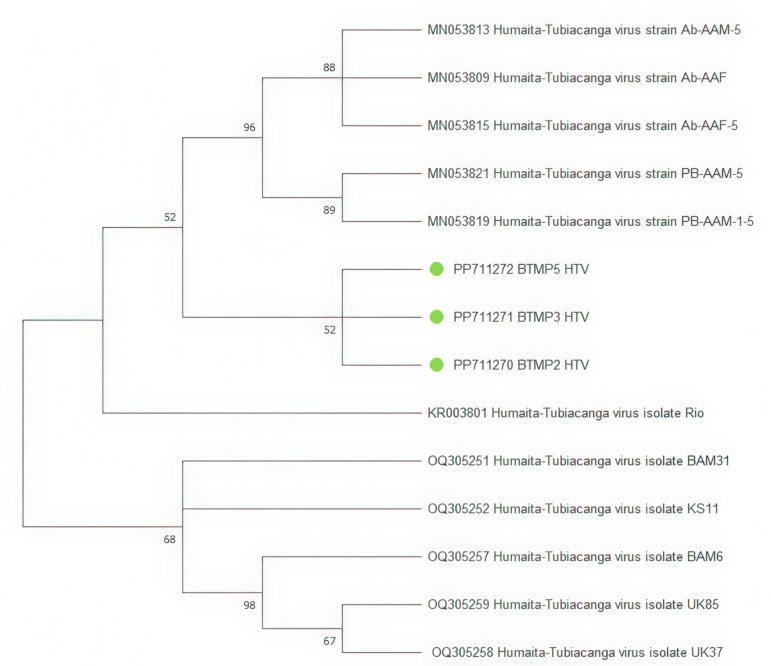
Phylogenetic tree of the *Humaita-Tubiacanga virus* (HTV) fragment. The evolutionary history was inferred using the Maximum Likelihood method and Kimura 2-parameter model [20]. Branches corresponding to partitions reproduced in less than 50% bootstrap replicates are collapsed. This analysis involved 14 nucleotide sequences. There were a total of 805 bp in the final dataset. The sequences obtained during the study are marked on the tree with green dots.

*Humaita-Tubiacanga virus* fragments were detected in six samples, and three sequences were used to construct the tree (Fig 7). On the phylogenetic tree, isolates BTMP2_HTV, BTMP3_HTV, and BTMP5_HTV are grouped together into a separate cluster aside from Guadeloupe samples (MN053813, MN053809, MN053815, MN053821, MN053819).

**Fig 8:**
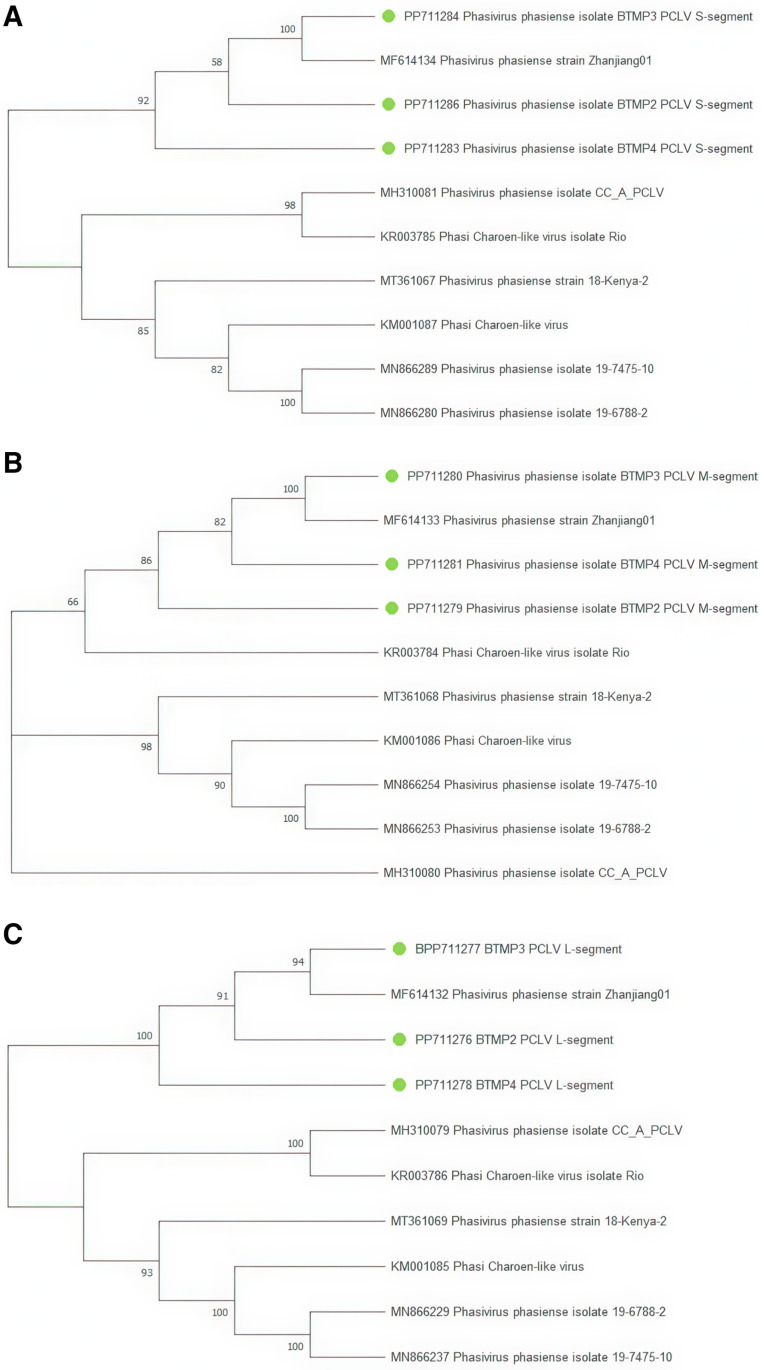
Phylogenetic tree of *Phasi Charoen-like phasivirus* (PCLV) genomic segments. Sequences obtained during the study are marked on the trees with green dots. Branches corresponding to partitions reproduced in less than 50% bootstrap replicates are collapsed. Panel 8a (S segment). The evolutionary history was inferred using the Maximum Likelihood method and Tamura-Nei model [22]. This analysis involved 10 nucleotide sequences. There were a total of 1335 bp in the final dataset. Panel 8b (M segment). The evolutionary history was inferred using the Maximum Likelihood method and Hasegawa-Kishino-Yano model [21**]. This analysis involved 10 nucleotide sequences. There were a total of 3770 bp in the final dataset. Panel 8c (L segment). The evolutionary history was inferred using the Maximum Likelihood method and Tamura-Nei model [**22]. This analysis involved 10 nucleotide sequences. There were a total of 6682 bp in the final dataset.

For the *Phasi Charoen-like phasivirus* (PCLV) (family *Phenuiviridae*), three near-complete sequences of the L-segment (RdRp), M-segment (glycoprotein), and S-segment (nucleocapsid) were assembled. Fragments of the virus were found in six samples, and three sequences of each segment were used to construct the tree (Fig 8). The constructed phylogenetic tree shows that the studied fragments of the L, M and S segments of PCLV are in the same clade with the sample obtained earlier in China (MF614132, MF614133, MF614134). Among PCLV strains, reassortment events were detected for M segment. In Fig 8b (segment M), a change in clade grouping is observed. The BTMP3_PCLV M-segment isolate and sample MF614133 (China) form a clade with the BTMP4_PCLV M-segment isolate. A change on branching is observed: isolate KR003784 (Rio) clusters with BTMP3_PCLV M-segment, BTMP2_PCLV M-segment, BTMP4_PCLV M-segment and MF614133, in contrast to the arrangement in L and S segments.

## 4. Discussion

Our study reports the first analysis of the RNA virus metavirome in *Aedes aegypti* mosquitoes in Vietnam. Altogether, the presence of classified viruses from 5 families was discovered. Additionally, *Kiowa virus* and *Guato virus* belong to unclassified taxa putatively from the *Chuviridae* family. The presence of certain viruses identified in this study are the first reports in Vietnam, specifically *Aedes aegypti totivirus*, *Aedes aegypti totivirus 2*, *Cell fusing agent virus*, *Guapiacu virus*, *Aedes flavivirus*, *Aedes aegypti To flavivirus-like*, *Verdadero virus*, *Chaq-like virus*, *Phasi Charoen-like phasivirus*, *Guadeloupe mosquito quaranja-like virus 1*, *Aedes aegypti To virus 1, Aedes aegypti To virus 2, Guato virus, Humaita-Tubiacanga virus, Kaiowa virus, Aedes binegev-like virus 1*, and *Aedes binegev-like virus 2*. The abundance of RNA viruses identified in the study is consistent with previous studies of the *Aedes aegypti* metavirome in other locations [23,24].

At the moment, ISVs are poorly understood, and some viruses are unclassified. Most ISVs are identified through metavirome studies. This study identified a wide range of ISVs, most of which do not have whole genome sequences and are represented in NCBI only by genomic fragments. Among those we identified, there are full genome sequences for *Aedes aegypti totivirus 1*, *Aedes aegypti totivirus 2*, *cell fusing agent virus*, *Aedes flavivirus*, *Phasi Charoen-like phasivirus*, *Aedes aegypti To virus 1*, and *Aedes aegypti To virus 2*.

Partial genomes of *Aedes aegypti totivirus* were identified in five pools in this study. This virus is a member of the *Totiviridae* family and the double-stranded RNA (dsRNA) virus group. The genomes of these viruses are 4700–6700 nucleotides in length, and the genome contains two large overlapping putative open reading frames (ORFs) [25]. RNA-dependent RNA polymerase (RdRp) is encoded by the 3’-proximal ORF, and capsid protein (Cap) is encoded by the 5’-proximal ORF.The highly structured 5’ end of the positive strand of the dsRNA genome is capless. Totiviruses contain a long 5′ untranslated region (5′ UTR) that functions as an internal ribosome entry site; have non-coding regions between ORFs [25,26]. On the constructed phylogenetic tree, isolates BTMP1_AaTV, BTMP3_AaTV, and BTMP4_AaTV formed a separate cluster. This indicates limited diversity of the virus in the territory of Binh Thuan, Central Vietnam.

CFAV is the first ISV reported and named after its characteristic cytopathogenic effect of cell fusion [27]. Phylogenetic analysis shows that the resulting isolate BTMP1_CFAV forms a cluster with isolate previously obtained from Cambodia (OR479688). Clustering of isolates BTMP1_CFAV and OR479688 from Cambodia indicates transmission of the virus between Cambodian and Vietnamese mosquitoes. This may indicate the possibility of the virus being introduced into different areas. Some findings suggest that CFAV may affect viruses such as ZIKV and DENV *in vitro* and *in vivo*. For example, results from Baidaliuk et al. (2019) indicate a decrease in transmission of ZIKV and DENV due to the presence of CFAV [28].

In this study, genomic fragments of the *Chaq-like virus* were assembled in three mosquito pools, belonging to the family *Partitiviridae*. Viruses belonging to the family *Partitiviridae* are known to infect a wide range of hosts, including plants and fungi. Some members of this genus, including the Chaq-like virus, have been recently discovered in arthropods [29,30]. The NCBI database contains sequences previously obtained in Nigeria and Mexico. Based on findings here and elsewhere, it can be assumed that the virus is widespread.

We made three assemblies for the L and M segments, as well as five for the S segment of *Phasi Charoen-like-phasivirus*. PCLV is one of the most common viruses in the Aedes aegypti virome. It belongs to the genus *Phlebovirus* of the family *Phenuiviridae* [31]. In the clade of PCLV strains, reassortment events were detected spanning the M segment. The reassortment phenomenon may serve as a source of genetic diversity in PCLV. Thus, much about the virus is still unknown, including details of its spread among mosquitoes. Recombination may indicate potentially high variability of the virus, which could lead to the formation of strains with new properties. In our results, PCLV was found to be one of the predominant ISVs in *Ae. aegypti*. The same results were previously observed for *Ae. aegypti* in Australia, Guadeloupe, South China, and Thailand [32–35].

The presence of the PCLV and HTV in the same mosquito pools was observed in in this study. Previously published data reported a possible beneficial co-infection between these viruses, enhancing the replication of arboviruses such as DENV and ZIKV in the *Ae. aegypti* mosquito population [9,25].

Despite the fact that all of the viruses detected in the mosquito pools studied belong to the group of insect-specific viruses, not much is known about their other potential hosts. Currently, there is evidence that ISVs infect insects and insect cells, but do not replicate in vertebrates or vertebrate cells [36]. Nevertheless, there is room for concern. It has been suggested that many pathogenic arboviruses acquired their dual-host diversification through adaptive evolution, through which an ancestral ISV gained the ability to infect vertebrates [37]. For these reasons, AaTV and AaTV2 represent potential emerging pathogens, yet the effects and outcomes of these viruses in mosquitoes are poorly understood [25].

A limitation of the study is that we did not find viruses with medical significance such as Dengue, Zika or Chikungunya which are major concerns as severe pathogens. Additionally, due to the high level of host RNA in samples, we did not manage to assemble whole genomes for the identified ISVs. Nevertheless, this is the first study to unveil the diversity of RNA viruses in Vietnam associated with *Aedes aegypti* mosquitoes, the main vectors of pathogens dangerous to human beings.

## Conclusion

This study highlights the need for next-generation sequencing to study the extensive diversity of viruses circulating in mosquitos in Vietnam. Further studies are needed to clarify possible interference of pathogenic viruses with ICV in *Aedes aegypti* mosquitoes. Additionally, deeper sequencing approaches may help with deciphering the whole genome of unclassified viruses detected in the study in order to clarify their taxonomic position. Experimental approaches are necessary to estimate the pathogenicity of these viruses.
